# Argon cold atmospheric plasma eradicates pathogens *in vitro* that are commonly associated with canine bacterial keratitis

**DOI:** 10.3389/fvets.2023.1320145

**Published:** 2024-01-09

**Authors:** Anne Helene Marx, Hilke Oltmanns, Jessica Meißner, Jutta Verspohl, Thomas Fuchsluger, Claudia Busse

**Affiliations:** ^1^Department of Small Animal Medicine and Surgery, University of Veterinary Medicine Hannover, Foundation, Hannover, Germany; ^2^Department of Pharmacology, Toxicology and Pharmacy, University of Veterinary Medicine Hannover, Foundation, Hannover, Germany; ^3^Department of Microbiology, University of Veterinary Medicine Hannover, Foundation, Hannover, Germany; ^4^Department of Ophthalmology, University of Rostock, Rostock, Germany

**Keywords:** plasma pen, non-thermal plasma, bacterial ulcer, antibacterial efficacy, MRSP

## Abstract

**Purpose:**

To investigate the antimicrobial effect of cold atmospheric plasma (CAP) on pathogens associated with canine bacterial keratitis.

**Materials and methods:**

*Pseudomonas aeruginosa*, *Staphylococcus pseudintermedius*, and *Streptococcus canis* strains, which were obtained from dogs with infectious keratitis, were subjected to testing. For each species, four isolates and a reference strain were cultivated on Columbia sheep blood agar and treated with the kiNPen Vet^®^ plasma pen from Neoplas GmbH, Greifswald, Germany. Various continuous treatment durations (0.5, 2, and 5 min) were applied, along with a 0.5-min treatment repeated four times at short intervals. These treatments were conducted at distances of 3 and 18 mm between the agar surface and the pen.

**Results:**

CAP treatment reduced bacterial growth in all three species. The most effective treatment duration was 5 min at 3 mm distance, resulting in inhibition zones ranging from 19 to 22 mm for *P. aeruginosa*, 26–45 mm for *S. pseudintermedius* and an overall reduction of bacterial growth for *Str. canis*. Inhibition zones were smaller with decreasing treatment duration and larger distance. Treatment times of 30 s repeated four times and 2 min showed comparable results. Treatment with argon alone did not lead to visible reduction of bacterial growth.

**Conclusion:**

Argon cold atmospheric plasma demonstrated a potent *in vitro* antimicrobial effect on *P. aeruginosa, S. pseudintermedius* and *Str. canis* strains with the latter showing the highest sensitivity.

## Introduction

Cold atmospheric plasma (CAP) is a relatively new tool in medicine that has gained much attention due to its biological effects on micro-organisms and living tissue – capable of stimulating wound healing processes in eucaryotic cells while eradicating prokaryotic cells (1, 2).

CAP is a partially ionized gas that consists of charged and neutral molecules, electromagnetic fields, free reactive molecules and is capable of emitting radiation in visible and ultraviolet range (3). CAP, also termed non-thermal plasma, operates at temperatures tolerated well by mammalian tissue. The most relevant plasma sources in medicine include dielectric barrier discharges and atmospheric pressure plasma jets (1, 4). The principle of the latter device is based on ionization of a working gas in between two electrodes. The components generated during this process are ejected from the pen by the gas flow of the working gas in form of a plasma jet (5). Exposure to this ‘plasma cocktail’ to biological targets generates reactive oxygen and nitrous species (ROS, RNS) which play a dominant role in the impact on the targets (6). ROS and RNS, which are important mediators in signaling processes, will stimulate redox-related processes within cells (7), or can cause cellular damage when reaching concentrations exceeding antioxidative capacities by inducing oxidative stress (8).

Current applications in the medical field mainly comprise therapy of chronic non-healing wounds (9, 10) and treatment of wound infections including multi-drug resistant infections (11). Identified mechanisms of antibacterial effects involve damage to the cell membrane or wall, to intracellular proteins, and DNA (1).

Newer fields of interest include cancer therapy (12, 13), dentistry (14) and ophthalmology (15). Many studies show that CAP can induce apoptosis of cancer cells *in vitro* (16–18). In dentistry, much attention was gained due to antibacterial efficacy of CAP (19–21). In human ophthalmology, CAP is an area of interest also due to its strong antimicrobial effect, demonstrated against various ocular pathogens, including bacteria (2, 22, 23), fungi (24), protozoa (25) and viruses (26), making it a potential device for the therapy of infectious keratitis. The safety of application of plasma jets was studied and did not cause significant damage to ocular tissue depending on dosage (15, 27). Reitberger *et al* published the first case series demonstrating the application of argon CAP in the therapy of infectious keratitis in people refractive to standard therapy. Adding argon CAP to the treatment regimen resulted in a substantial improvement of disease after the third or fourth application (15).

Infectious ulcerative keratitis is a common disease in small animals (28). Many corneal pathogens have been identified to be associated with the disease, the majority of isolated bacteria however belong to *Pseudomonas aeruginosa*, *Staphylococcus pseudintermedius* and *Streptococcus canis* species (29–36). The therapy of choice is topical antimicrobial treatment (37, 38). If treatment fails to control infection, for instance either due to incorrect choice of the antimicrobial agent or the involvement of a multidrug resistant bacterial isolate, the disease can rapidly progress with sight and globe threatening consequences (28, 37). *Staphylococcus* species isolated from dogs with infectious keratitis frequently include methicillin-resistant isolates (39). Rising concerns about the development of antimicrobial resistances among canine corneal pathogens against commonly used antibiotic agents (30, 38, 40) and the restrictive legal use of antibiotics by legislation within the European Union (41), highlight the importance of establishing alternative antimicrobial treatment modalities. We therefore investigated the antibacterial efficacy of argon CAP against corneal pathogens isolated from canine patients with bacterial keratitis.

## Materials and methods

### Bacterial isolates

Clinical isolates were collected by a laboratory for clinical diagnostics (LABOKLIN GmbH & Ko KG, Bad Kissingen, Germany). Swap samples were obtained from canine patients diagnosed with bacterial ulcerative keratitis for the purpose of species identification and antimicrobial resistance testing. Four field isolates were chosen from each of the following bacteria: *Staphylococcus pseudintermedius* (SP), *Streptococcus canis* (SC) and *Pseudomonas aeruginosa* (PA). For each bacteria a reference strain was included in the study (DSM 20373, DSM 20716, DSM 10880). Inclusion criteria for all isolates included the presence of a stromal ulcer at the time of sampling and available antimicrobial susceptibility profiles, shown in Table 1. We included two methicillin-resistant and two non-methicillin-resistant isolates of SP. In the case of PA included isolates were either resistant or susceptible to difloxacin and orbifloxacin, each group was also represented by two isolates. Antimicrobial susceptibility patterns among included SC isolates were similar.

**Table 1:** Antimicrobial susceptibility of clinical isolates.

Isolate	1	2	3	4	5	6	7	8	9	10	11	12	13	14	15	16	17	18	19	20	21	22	23	24	25	26	27	28	29	30	31
PA1	S	R	S	S	R	R	S	R	R	R	R	R	S	S	S	R	R	R	S	R	R	R	R	R	R	S	R	S	R	S	R
PA2	S	R	S	R	R	R	S	R	R	R	R	R	S	S	S	R	R	R	S	R	R	R	R	R	R	S	R	S	R	I	R
PA3	S	R	S	R	R	R	S	R	R	R	R	R	S	S	S	R	R	R	S	R	R	R	R	R	R	S	R	S	R	I	R
PA4	S	R	S	S	R	R	S	R	R	R	R	R	S	S	S	R	R	R	S	R	R	R	R	R	R	S	R	S	R	S	R
SP1	S	S	S	S	S	S	S	S	S	S	S	S	S	S	S	S	S	S	R	S	S	S	S	S	S	S	S	S	S	S	S
SP2	S	S	S	S	S	S	S	S	S	S	S	S	S	S	S	S	S	S	R	S	S	S	S	S	S	S	S	S	S	S	S
SP3*	I	R	R	R	R	S	I	R	R	R	S	R	R	S	S	R	R	R	R	R	R	R	S	R	R	R	R	I	R	R	S
SP4*	I	R	S	S	R	S	I	R	R	R	R	S	S	S	S	R	R	R	R	R	R	R	S	R	R	S	R	S	R	S	R
SC1	R	S	S	S	S	R	R	S	S	S	S	R	S	R	R	S	S	S	R	S	S	S	S	S	S	S	S	S	S	S	S
SC2	R	S	S	S	S	R	R	S	S	S	S	S	S	R	R	S	S	S	R	S	S	S	S	S	S	S	S	S	S	S	S
SC3	R	S	R	S	S	R	R	S	S	S	S	S	S	R	R	S	S	S	R	S	S	S	S	S	S	S	S	I	S	R	S
SC4	R	S	I	I	S	R	R	S	S	S	S	S	I	R	R	S	S	S	R	S	S	S	S	S	S	I	S	I	S	I	S

(1) Tobramycin, (2) Cefquinome, (3) Marbofloxacin, (4) Difloxacin, (5) Spiramycin, (6) Fusidic acid, (7) Gentamicin, (8) Cephalexin, (9) Amoxicillin & clavulanic acid., (10) Tetracycline, (11) Chloramphenicol, (12) Trimethoprim/Sulfamethoxazole (13) Enrofloxacin, (14) Neomycin/Framycetin, (15) Kanamycin, (16) Cephoperazone, (17) Penicillin-G, (18) Amoxicillin, (19) Polymyxin B/Colistin, (20) Doxycycline, (21) Erythromycin, (22) Clindamycin, (23) Nitrofurantoin, (24) Lincomycin, (25) Ampicillin, (26) Ofloxacin, (27) Cefovecin, (28) Pradofloxacin, (29) Cefoxitin, (30) Orbifloxacin, (31) Florfenicol; *indicates methicillin-resistant strains.

### Plasma source

Experiments were conducted by using a modified kINPen^®^Vet manufactured by Neoplas ^®^ GmbH, Greifswald, Germany, which consists of an operating device, a gas supply line and the hand-pen. Plasma generation takes place in the hand-pen in-between electrodes with a high-frequency voltage (1.0 MHz, 2–3 kV) when argon gas at a flow rate of 4–6 SLM is applied. This results in plasma expelled in form of a plasma jet, visible as a beam of approximately 1 cm length.

**Figure 1:**
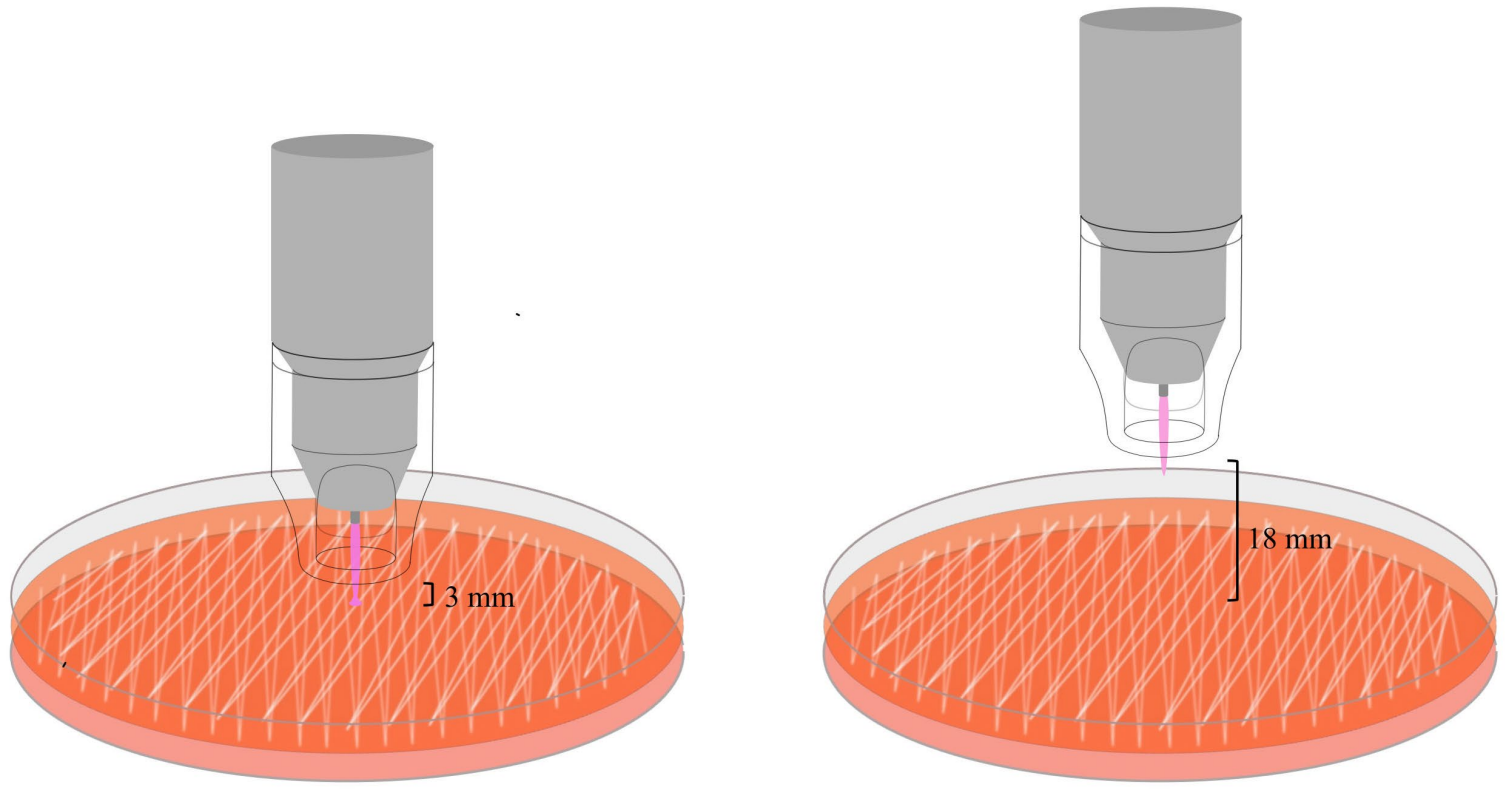
The schematic illustration shows the exposure of bacteria on agar plates with the plasma pen at a distance between the spacer and the agar surface of either 3 or 18 mm. Note the conducting effect of the plasma beam in the left image – the intensity of the light beam increases and the light spreads over a small portion of the treated area. This happens when the plasma directly hits a conductive surface.

The device was equipped with an additional switch to interrupt plasma emission. Plasma is generated inside the pen during ionization of argon gas with a high-frequency voltage and is then pink spot beam of about 1 cm in length at the tip of the pen. A transparent spacer that keeps a minimum distance of 7 mm between the tip of the pen and the treated surface was placed (Figure 1).

The gas flow rate was adjusted to 4.1 SLM at a constant gas pressure of 2.5 bar (recommended pressure by the manufacturer is 2–3 bar). We decided to decrease the gas flow rate as much as the device allowed because the gas flow rate of 5 SLM that was used in human patients (15) resulted in desiccation of the agar surface (observations during preliminary experiments). Modification of the device allowed turning off the production of plasma without modifying the gas flow rate.

### Temperature measurement

To evaluate whether to expect thermal effects on bacterial growth, the temperature of the plasma jet was measured at the tip of the plasma jet via a contact k-type element of a digital thermometer (MESTEK 800C^®^). The contact element was exposed to the tip of the plasma jet for 5 min and measurements were taken every minute.

### Experiments

Bacterial isolates were routinely cultured on Columbia blood agar (with 7% Sheep Blood; OXOID) for an incubation period of 20–24 h at 37°C. A bacterial suspension was prepared using an optical densitometer (McFarland = 1.0). A further 10-fold dilution of the suspension was performed to culture isolates for the experiments since this resulted in a uniform growth of bacteria on the agar plates. The same suspension was used for all experiments with the same isolate to eliminate falsifications resulting from variations in bacterial concentration. Two serial dilutions were made for each suspension by spreading 100 μL of suspension onto agar plates in duplicates.

Inoculated plates were treated with either argon gas or plasma for 0.5, 2, 5, and 4 × 0.5 min (with a pause of 0.25 min in between, which is the approximate time needed to apply a lubricant eye drop). Treatment times were selected based on results from the study by Reitberger et al. (15), showing antimicrobial effects following treatment of human corneal pathogens for 2, 5, 7 and 10 min using the same device. We considered a treatment of the corneal surface of small animals for 7 and 10 min as clinically not applicable with the current device and did not include treatments exceeding 5 min. Furthermore, in the previously mentioned study a treatment time of 0.5 min was selected for the application on *in vivo* experiments. We therefore decided to include 0.5 min in our protocol. An overview of the treatment protocol can be seen in Figure 1.

The plasma pen was stationary and the distance between the spacer and the plate was set to 3 or 18 mm. A distance of 3 mm was the largest distance that still resulted in contact between the plasma beam and the surface, creating a conductive mode. We wanted to investigate the effects of a larger distance that would avoid direct contact of the jet potentially reducing the effects of gas flow on corneal tissue. Preliminary studies (unpublished) did not show consistent results with the previously used distance of 5 cm. A distance of 2 cm resulted in less drying effects (unpublished data) and for technical reasons a distance of 18 mm was used. As a control, all protocols were repeated with argon gas alone. All agar plates were incubated for 19–21 h at 37°C. Serial dilutions were determined by counting colony forming units (CFUs). Treated plates were evaluated for inhibition zones that were measured using a stainless steel vernier caliper (mm). In the case of circular shaped inhibition zones the diameter was measured, in irregular shaped zones two measurements were taken – one at the widest and one at the smallest width of the zone. In the case of ill-defined margins, zones where a reduction of bacterial growth was visible were measured. Overall reduction of bacterial growth was recorded, but not further classified. Experiments were performed in duplicates and results are given as mean values.

## Results

### Temperature measurement

The results of temperature measurements showed values between 39.8 and 40.1°C throughout the treatment period.

### Treatment of isolates

**Figure 2:**
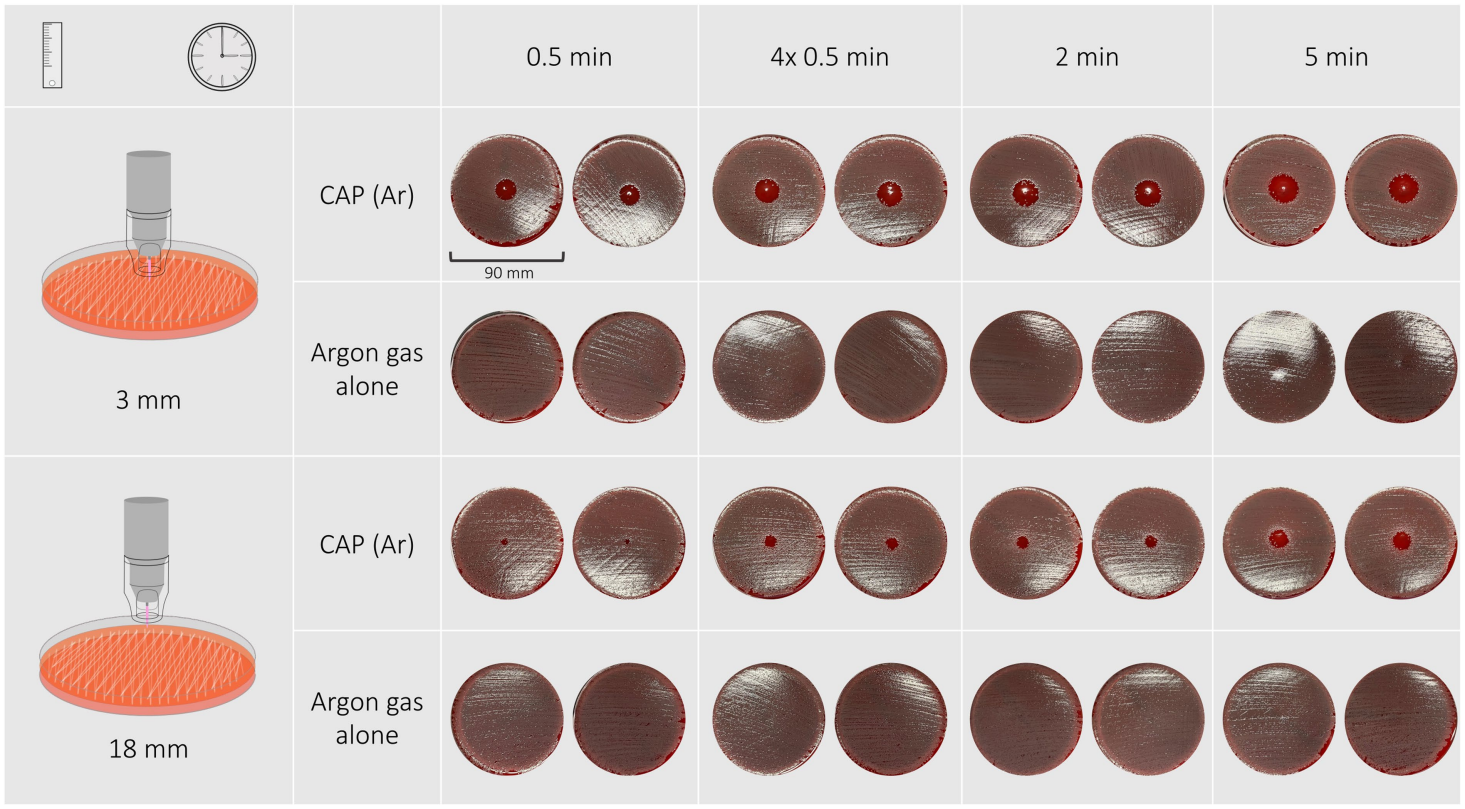
The inhibition zones of a *Pseudomonas aeruginosa* isolate after exposure to CAP for 30 s, 4 × 30 s, 2 min, 5 min at a distance of 3 mm are circular and have distinct edges. None of the treatments with argon gas alone resulted in the formation of inhibition zones.

**Figure 3:**
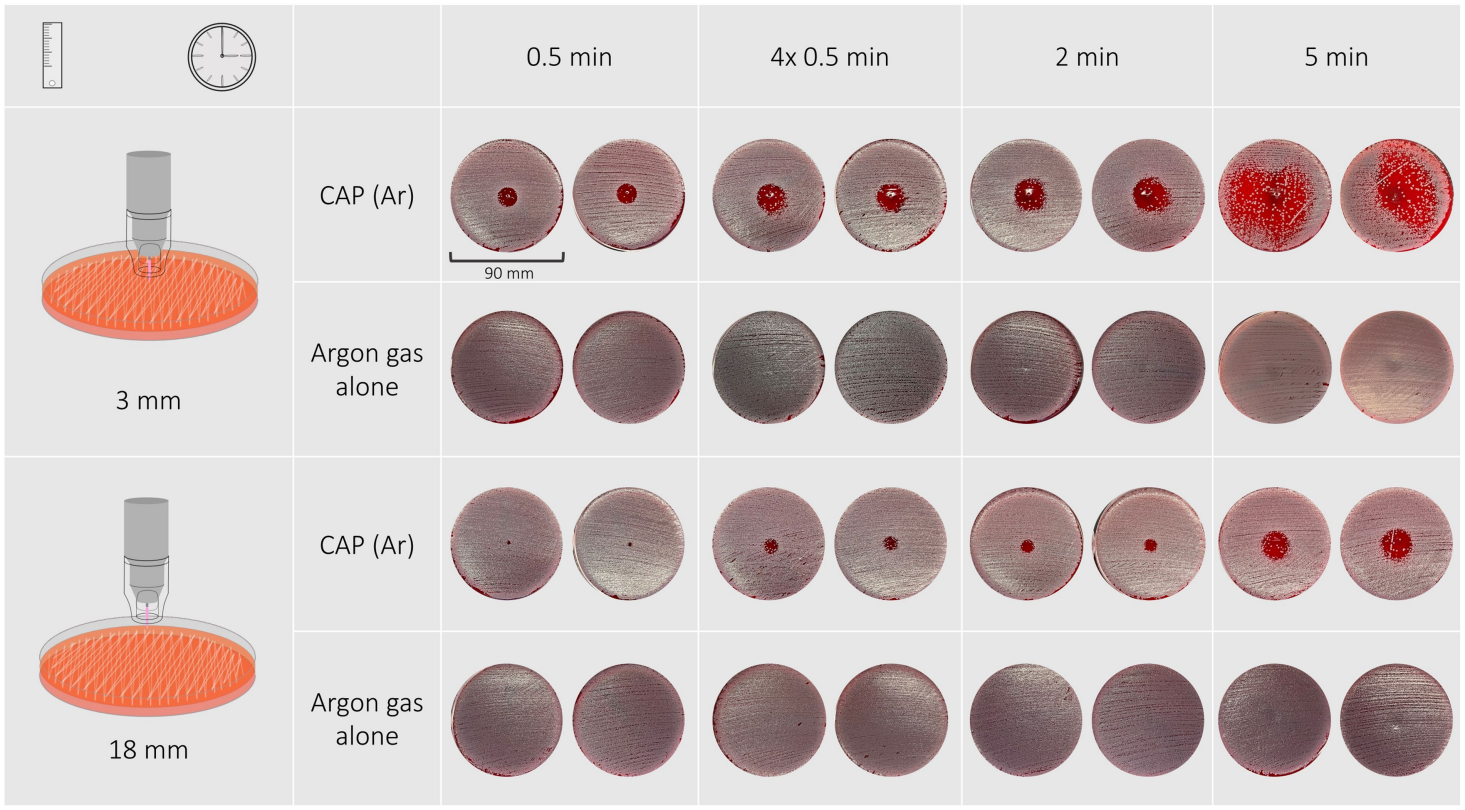
Inhibition zones of a *Staphylococcus pseudintermedius* isolate after exposure to CAP for 30 s, 4 × 30 s, 2 min, 5 min at a distance of 3 mm are circular and the edges become ill-defined with longer treatment duration.

All treatment protocols showed a clear antimicrobial effect visible as an inhibition zone or an overall reduction of bacterial growth on the treated agar plates. No antimicrobial effect was noticed on plates solely treated with argon gas. Inhibition zones varied in shape and definition of margins between bacterial species, treatment duration and distance of the spacer to the treated surface. Treatment of *P. aeruginosa*, *S. pseudintermedius* and *S. canis* isolates resulted in inhibition zones with either a circular shape with distinct or indistinct margins, or an irregular shape or overall reduction of bacterial growth, respectively. Inhibition zones can be seen in Figures 1–3, their measurements can be taken from Table 2.

**Table 2:** Size of inhibition zones of *P. aeruginosa* isolates (PA1-4), *S. canis* (SC1-4), *S. pseudintermedius* (SP1-4) and their reference strains (PAR, SCR, SPR) given in mm.

Time (min)	0.5		2 (4 × 0.5)		2		5	
Distance (mm)	3	18	3	18	3	18	3	18
PAR	16	5	18	10	19	10	22	14
PA1	11	5	18	10	19	10	22	15
PA2	14	6	16	10	17	9	19	14
PA3	15	5	17	9	18	9	21	16
PA4	16	6	18	11	18	11	21	18
SPIR	14	4	25	14	22	14	45	28
SPI1	14	3	21	9	21	13	28	19
SPI2	15	3	20	10	22	9	37	20
SPI3	14	3	19	10	20	10	26	21
SPI4	15	3	21	10	24	10	43	21
SCR	18	4	42 × 49	21	42 × 57	22	OR	50 × 56
SC1	19	10	41 × 46	28	40 × 46	29	OR	62 × 70
SC2	17	6	49 × 4	23	50 × 57	21	OR	54 × 63
SC3	18	8	42 × 45	22	48 × 73	24	OR	52 × 60
SC4	18	5	37 × 47	21	44 × 50	20	OR	45 × 50

OR = overall reduction of bacterial growth.

### Pseudomonas aeruginosa

Inhibition zones of all *P. aeruginosa* isolates and the reference strain were circular with distinct margins as shown in Figure 2. A treatment duration of 5 min at a distance of 3 mm had the strongest antimicrobial effect and resulted in inhibition zones ranging from 19 to 22 mm (reference strain: 22 mm). The least effective treatment duration was 30 s at a distance of 18 mm that resulted in zones ranging from 5 to 6 mm (reference strain: 5 mm). 30-s exposure time at a distance of 3 mm resulted in inhibition zones ranging from 11 to 16 mm (reference strain: 16 mm), 2-min exposure time at the same distance resulted in inhibition zones of 18–19 mm (reference strain: 19 mm). Exposing bacteria to either 2 min or 30 s repeated four times resulted in similar inhibition zones ranging from 17–19 mm and 17–18 mm with 3 mm distance and from 9–11 mm and 9–11 mm with 18 mm distance, respectively.

### Staphylococcus pseudintermedius

Morphology of inhibition zones of *S. pseudintermedius* isolates and reference strain varied between different exposure times and distances (Figure 3). A treatment duration of 30 s and 2 min resulted regardless of distance in a circular inhibition zone with distinct margins. A treatment duration of 5 min at a distance of 3 mm resulted in either a circular inhibition zone with indistinct margins or an irregular shaped inhibition zone. Treatment duration of 5 min at a distance of 18 mm led to circular shaped zones with indistinct margins.

A treatment duration of 5 min at a distance of 3 mm had the most effective antimicrobial effect and resulted in inhibition zones ranging from 28 to 43 mm (reference strain: 45 mm). The least effective treatment duration was 30 s at a distance of 18 mm that resulted in zones measuring 3 mm (reference strain: 4 mm). 30-s exposure time at a distance of 3 mm resulted in inhibition zones measuring 14 mm (reference strain: 14 mm), 2-min exposure time at the same distance resulted in inhibition zones of 20–24 mm (reference strain: 22 mm). Exposing bacteria to either 2 min or 30 s repeated four times resulted in similar inhibition zones ranging from 20–24 mm and 21–25 mm with 3 mm distance and from 9–14 mm and 9–14 mm with 18 mm distance, respectively.

### Streptococcus canis

**Figure 4:**
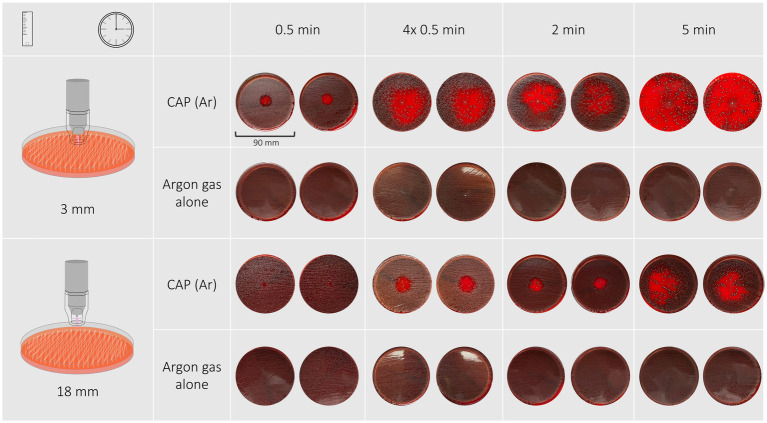
Shown are the inhibition zones of a *Streptococcus canis* isolate after exposure to CAP for 30 s, 4 × 30 s, 2 min, 5 min at a distance of 3 mm. With short treatment the zones were circular with undefined edges, with longer treatment times the shapes became irregular. A 5-min treatment resulted in a reduction of bacterial growth on the entire agar plate.

Response of *S. canis* isolates and the reference strain to cold plasma treatment was most variable among bacterial species. Inhibition zones were either circular with distinct or indistinct margins, irregular shaped or were not measurable due to an overall reduction in bacterial growth (Figure 4). Treatment for 30 s with both distances resulted in inhibition zones with distinct margins, 2 min and four times 30 s both at 18 mm distance resulted in circular shaped zones with either distinct or indistinct margins. Treatment duration of 2 min and four times 30 s at 3 mm distance resulted in irregular shaped inhibition zones. A 5-min treatment duration resulted in overall reduction of bacterial growth on the plate in the case of 3 mm distance, or in an irregular shaped inhibition zone in the case of 18 mm distance.

The most effective antimicrobial effect was achieved with a treatment duration of 5 min at a distance of 3 mm which resulted in overall reduction of bacterial growth. The least effective treatment duration was 30 s at a distance of 18 mm that resulted in zones ranging from 5 to 10 mm (reference strain: 4 mm). 30-s exposure time at a distance of 3 mm resulted in inhibition zones ranging from 18 to 19 mm (reference strain: 18 mm), 2-min exposure time at the same distance resulted in inhibition zones with a minimal and maximal width ranging from 40 to 50 mm (reference strain: 41 mm) and 46–73 mm (reference strain: 57 mm), respectively. Exposing bacteria to either 2 min or 30 s repeated four times at a distance of 3 mm resulted in variably irregular shaped inhibition zones with a minimal and maximal width ranging from 40–73 mm and 37–54 mm, respectively. Treatment durations of 2 min and 30 s repeated four times at 18 mm distance resulted in inhibition zones ranging from 20–29 mm and 21–28 mm, respectively.

### Comparison of bacterial species and treatments

Increasing treatment duration and reducing distance to the spacer resulted in larger inhibition zones in all species, thus the strongest antimicrobial effect was detected after a treatment duration of 5 min at 3 mm distance, the least after a treatment duration of 30 s at 18 mm distance.

A treatment duration of 30 s at a distance of 3 mm resulted in inhibition zones of up to 16, 15 and 19 mm in the case of *P. aeruginosa, S. pseudintermedius* and *S. canis* isolates, respectively. These zones were still larger compared to those resulting from a 2-min treatment duration at 18 mm distance in *P. aeruginosa* and *S. pseudintermedius* isolates and were even similar to those resulting from a 5-min treatment at 18 mm distance in the case of *P. aeruginosa* isolates.

## Discussion

To the best of our knowledge, this is the first study evaluating the *in vitro* antibacterial efficacy of argon cold atmospheric plasma against common corneal pathogens involved in canine bacterial keratitis. All clinical isolates of *Pseudomonas aeruginosa*, *Staphylococcus pseudintermedius* and *Streptococcus canis* were susceptible to the exposure to argon CAP.

### Sensitivity patterns of different bacteria

The sterilizing effects of CAP on various bacterial species are well reported (2, 11, 15, 19–23, 42–46), there are however differences in sensitivity patterns among species (15, 42, 43, 47–50). Different levels of sensitivity were also seen in our study presenting as variably sized and shaped inhibition zones. Isolates of *S. canis* showed the largest inhibition zones and bacterial growth was inhibited within areas reaching far over the focally treated spot. Inhibition zones of *S. pseudintermedius* and *S. canis* isolates showed ill-defined margins. This could result from a lower concentration of plasma components at the periphery of the plasma jet as they are highly reactive in atmospheric air (3). Inhibition zones of *P. aeruginosa*, in contrast, had well-defined margins and were circular shaped. We therefore suspect that eradication of *P. aeruginosa* requires exposure to higher concentrations of plasma, which is supported by a previous study comparing susceptibility of *P. aeruginosa* to *S. pseudintermedius* isolates from skin infections in dogs (50). Current *in vitro* studies show contradictory results when it comes to the difference in susceptibility of Gram positive and Gram negative species (15, 46, 47, 51, 52). Some studies report a higher sensitivity of Gram negative compared to Gram positive bacteria (51, 52). As cellular membrane damage is considered to play a major role in the mechanism of antimicrobial efficacy of CAP it is postulated that cell wall morphology contributes to the difference in sensitivity (51, 52). Results from our study do not support this theory. *S. canis* isolates were most susceptible in our study which may emphasize that cellular membrane damage is not the only mechanism of antibacterial efficacy (53). This finding correlates with a previous study that showed increased intracellular reactive oxygen radicals in Gram positive compared to Gram negative species following exposure to CAP (48). The exact reason for that remains unknown.

As we postulate that the maximum treatment duration at a time is 30 s – for reasons discussed later – we included an interval treatment of 2 min consisting of 4 intervals with 15 s in between. The results from that protocol showed similar results as those from protocols using 2 min of full exposure in all bacterial species. We therefore do not expect a decrease in antibacterial efficacy of interval treatment in the case of a 2-min treatment duration.

Complex and incompletely understood biochemical and biophysical reactions of the different pathogens to components of CAP cause different sensitivity patterns between bacterial species, *S. canis* isolates were most susceptible to the exposure to CAP in our study.

### Efficacy against multi-drug-resistant isolates

CAP is highly effective in eradicating multi-drug resistant bacteria *in vitro* and *in vivo*, such as isolates of methicillin-resistant *Staphylococcus aureus* (MRSA) (44, 54, 55). This is in concordance with findings in our study showing that the treatment of two methicillin-resistant isolates of *S. pseudintermedius* achieved very similar results compared to methicillin-sensitive isolates. The development of multidrug resistances against CAP seems unlikely since multiple components are involved in antibacterial efficacy (42, 53, 56, 57). Furthermore, CAP may even increase efficacy of antibiotic agents as its previous exposure increases sensitivity of *methicillin-resistant Staphyloccus aureus* strains (MRSA) to antibiotics (58). In addition to MRSA, eradication of methicillin-resistant *Staphylococcus pseudintermedius* isolates may also be a potential goal of the application of CAP.

### Factors influencing antimicrobial efficacy

Antimicrobial outcomes depend on a number of variable factors, including the choice of plasma source, power supply, working gas and the dosage of CAP. The type of plasma sources is influencing the efficacy because they may use variable techniques of plasma generation that produce plasma of different composition and quantity (1, 42). The principle of plasma generation of plasma jets is based on ionization of a working gas which flows in between two electrodes. The type of working gas, for instance argon or helium gas, influences antimicrobial efficacy, as concentration of reactive oxygen radicals in plasma differs depending on the type of gas (59, 60). It is reported to be higher in plasma generated with argon gas compared to helium gas (59, 60). The device in our study operates with argon gas which has several advantages. These include a good tissue tolerance (61), its natural occurrence in the atmosphere (62) and simple extraction by air liquefaction (63). Another advantage of argon in research is its ‘inert state’ which enabled us its use in our control experiments which verified that argon gas does not have an antibacterial effect itself. Helium gas, in contrast, is non-renewable and its recycling is far too low to cover the current supply, causing scarcity and rising prices in the long run (64). Another factor influencing antimicrobial efficacy of CAP is the addition of different concentrations of oxygen to the working gas (46). Investigating the effect on pathogens by adding oxygen to argon gas was not within the scope of the current study. The dosage of plasma also has a significant impact on antimicrobial efficacy (15, 42, 48–50). This correlates with results from our study showing that efficacy was stronger with increased exposure time and shorter distance to the treated surface.

The high number of factors potentially influencing *in vitro* antimicrobial efficacy makes it very difficult to compare antibacterial efficacy from different studies (1). The results seen in our study may therefore not be reproduced using a different plasma source, working gas or experimental design.

### Limitations of the study

Limitations of this study were a low number of tested isolates and the inclusion of only three different bacterial species from the ocular surface. Expanding experiments to other corneal pathogens in dogs is necessary to further investigate the antibacterial spectrum of argon cold atmospheric plasma.

### Future research

The potential use of CAP generated by the kINPenVET^®^ in the therapy of bacterial keratitis in canine patients requires future research. This should focus on safety for ocular tissue, penetration depth of CAP into corneal tissue and its *in vivo* efficacy against corneal pathogens.

Examples for potential mechanisms of damage to ocular tissue accompanying the exposure to CAP may include interactions with reactive species, exposure to UV light (65) or desiccation of the ocular surface by high gas flow rates (15).

Current research focusing on the safety of CAP for human corneal tissue is promising, as it shows that CAP does not result in harmful effects on *in vitro* cultures of different ocular cells if applied for up to 5 min (15, 27). CAP has even been shown to increase viability of *in vitro* corneal limbal epithelial cells following an exposure to argon CAP for up to 2 min. Exposure for 5 min did not influence viability, while a decrease was seen after 10 min (15). Free radicals were long considered toxic due to their high reactivity (66), recent studies however have shown that intracellular processes rely on the presence of free radicals (1). Their presence most likely plays the dominant role in stimulatory effects on cells resulting in increased cell migration (67) and proliferation (68). Furthermore, in chronic wounds important processes relying on radicals are impaired and can be re-stimulated by increasing intracellular concentrations of reactive species (1). Corneas treated for up to 5 min did not show histopathological changes in studies using different devices (15, 23, 45, 69, 70). The rate of corneal epithelization in rabbits was not affected by the application of CAP for 2 min (69). Ultraviolet light (UV) is one of the components of CAP with well reported toxic effects to corneal tissue (71). The risk of UV-induced damage during exposure to CAP is considered to be low due because emission of UV light during generation of CAP is low (72) and there was no evidence for UV-induced damage to conjunctival fibroblasts and corneal specimens after exposure for up to 5 min in previous studies (2, 27). Desiccation of the ocular surface was observed after application of the kINPen MED plasma pen (Neoplas GmbH^®^) to the cornea of human patients for 30 s (15). This is the consequence of a high gas flow rate of the working gas which is needed for adequate plasma concentrations and aids in the cooling of the device (personal communication with Neoplas GmbH^®^). We decided to reduce the gas flow rate to 4.1 SLM due to technical reasons, it can however be reduced up to a flow rate of 2 SLM according to the manufacturer. We postulate that lubrication of the patient’s eye during therapy may be needed every 30 s – interrupting the treatment is less likely to interfere with antimicrobial efficacy as discussed in the previous section.

Research on penetration depth of CAP into corneal tissue is scarce. One study using a corneal stromal tissue model showed that the antimicrobial effect on *E. coli* was evident in a depth of up to 200 μm (73). CAP in this study was however generated by a different plasma source.

Studies investigating the antimicrobial efficacy of CAP on ocular pathogens under ex or *in vivo* conditions exist. For instance, CAP was used to successfully treat experimentally infected *ex vivo* (15, 24, 27, 45) and *in vivo* corneas (22) of people, rabbits and pigs. Naturally occurring corneal infections in people were treated with CAP using the kINPen MED^®^ (15). In all studies treatment with CAP alone or as an addition to standard therapy was efficient to control infection and resulted in healing of corneal ulceration.

Based on current literature, the application of CAP in patients with corneal infections may be safe and in theory efficient to treat the infection, however, randomized, clinical studies with control groups are needed to confirm its efficacy in the therapy of bacterial keratitis. We encourage research on all these aspects in companion animals as they are currently lacking.

## Conclusion

We conclude that argon cold atmospheric plasma demonstrates a potent *in vitro* antibacterial effect on *P. aeruginosa, S. pseudintermedius* and *Str. canis* strains, bacteria most commonly found in canine bacterial keratitis. It is also capable of eradicating multi-drug resistant bacteria, including methicillin-resistant *S. pseudintermedius* isolates and may increase efficacy of antibiotics if used in combination (58). If future research confirms our results and the safety of its application, cold atmospheric plasma could be a promising alternative or adjuvant to antibiotics on the ocular surface in dogs. It could therefore aid in the reduction of antibiotic consumption which is in line with the one health approach (74).

## Data availability statement

The original contributions presented in the study are included in the article/supplementary material, further inquiries can be directed to the corresponding author.

## Ethics statement

Ethical approval was not required for the study involving animals in accordance with the local legislation and institutional requirements because the bacterial isolates used in the study were previously obtained from clinical patients for species identification and susceptibility testing (reasons unrelated to the study).

## Author contributions

AM: Investigation, Methodology, Visualization, Writing - original draft, Writing - review & editing. HO: Methodology, Resources. JM: Methodology, Resources. JV: Methodology, Resources. TF: Conceptualization. CB: Conceptualization, Methodology, Supervision, Visualization, Writing - review & editing.
